# New and noteworthy records of *Myriolecis* (Lecanoraceae, lichenized Ascomycota) in the arid regions of northwestern China

**DOI:** 10.3897/mycokeys.133.186955

**Published:** 2026-06-08

**Authors:** Tursunay Payzulla, Guldiyar Adil, Reyim Mamut

**Affiliations:** 1 College of Life Sciences and Technology, Xinjiang University, Huarui Road, Urumqi, 830017, Xinjiang, China College of Life Sciences and Technology, Xinjiang University Urumqi China

**Keywords:** *Lecanora dispersa* group, lichen taxonomy, molecular phylogeny, new taxon, xanthones

## Abstract

*Myriolecis* represents one of the richest genera in species diversity within Lecanoraceae. In this study, approximately 700 specimens collected from high-altitude, temperate continental arid-climate regions in northwestern China, including the Kunlun Mountains, the southern Tianshan Mountains, and the Karakoram Range, were examined. A total of six species were identified, among which three are described as new to science: *Myriolecis convexa*, *M. incisa*, and *M. kunlunica*; and three are newly recorded in China: *M. fugiens*, *M. mons-nivis*, and *M. wetmorei*. All species were identified based on morphological, anatomical, and molecular data. Phylogenetic analyses using internal transcribed spacer (ITS) and mitochondrial small subunit (mtSSU) sequences confirm the placement of these species within *Myriolecis* and support the current species delimitations. Detailed morphological descriptions, illustrations, distribution data, chemical information, and ecological notes are provided for each species, along with comparisons with morphologically similar and phylogenetically related species within the genus. A dichotomous key to 22 species of *Myriolecis* from China is presented.

## Introduction

*Myriolecis* Clements (formerly the *Lecanora dispersa* group) has a worldwide distribution but is most diverse in temperate to arctic-alpine regions of the Northern Hemisphere. It is characterized by an endolithic or epilithic white thallus; apothecia with a mostly white or light-colored rim; secondary chemistry usually with a wide range of depsides and depsidones, terpenoids, or xanthones; and usually growing on calcareous substrates (Poelt et al. 1995; Cannon et al. 2022). It is considered one of the most taxonomically challenging genera, exhibiting considerable intraspecific variability and species complexes within *Lecanora* (Laundon 2003; Śliwa 2007). Laundon (2003) briefly discussed the entire *L. dispersa* group and provided suggestions for some taxonomic and nomenclatural changes. Śliwa (2007) conducted a detailed investigation into the North American *L. dispersa* group, covering aspects such as history, thallus morphology, apothecial morphology and anatomy, chemistry, and ecology. The study identified the members of this group and provided foundational guiding materials for subsequent research on the group. Śliwa et al. (2012) evaluated the delimitation of selected morphospecies within the *Lecanora dispersa* group and reconstructed relationships among them using phylogenetic inferences (Bayesian, maximum likelihood (ML), and maximum parsimony (MP) inferences) based on DNA sequences from the internal transcribed spacer (ITS) region. This was the first attempt toward a modern taxonomy of the *L. dispersa* group. Zhao et al. (2016) found that only a few of the informally recognized groups were phylogenetically well delimited and recognized them as distinct genera (*Myriolecis*, including *Arctopeltis* Poelt for the *Lecanora dispersa* group). Recently, Kondratyuk et al. (2019), based on DNA sequence data from four loci, suggested reinstating *Polyozosia* A. Massal. as an older name for *Myriolecis* and accepting *Glaucomaria* M. Choisy as a genus separate from *Lecanora*. In summary, through the efforts of lichenologists worldwide, this lichen genus has now been established as a monophyletic lichen group with clearly defined boundaries, distinct diagnostic characteristics, and a stable composition of species. Currently, 50 species of *Myriolecis* have been accepted by Index Fungorum (http://www.indexfungorum.org/, accessed on 24 November 2025).

Information on lichen taxonomic research in the Kunlun-Karakoram Mountains is limited. During the 2003–2022 period, lichen specimen collections were conducted in the Kunlun Mountains, the southern Tianshan Mountains, and the Karakoram Range within China, as well as in the distribution areas of *Myriolecis* species that had been reported by Magnusson (1940). These collection areas encircle the western edge of the Tarim Basin and also include parts of the massive tectonic system of the Qinghai-Tibet Plateau. Consequently, high altitude, intense erosion, and active tectonic activity are their common characteristics. The rock composition of this region profoundly reflects its complex tectonic history. Its components are limestone, mudstone, sandstone, conglomerate, and granite. These rocks exhibit a low degree of consolidation, are relatively soft, and are highly susceptible to weathering and erosion, which has led to the formation of typical folded mountain landscapes and also serves as important habitats for crustose lichen diversity. The entire region falls under a temperate continental arid climate, where vegetation exhibits distinct xeric and alpine characteristics with remarkably pronounced vertical zonation. Desert vegetation predominates, with dominant species including super-xerophytic shrubs and semi-shrubs such as *Tamarix* spp., *Haloxylon ammodendron* (C.A.Mey.) Bunge, *Alhagi sparsifolia* Shap., and *Ephedra* spp. In locally moist areas, particularly on north-facing or partially shaded slopes of the Tianshan and Kunlun Mountains, fragmented patches of *Picea schrenkiana* Fisch. et Mey. forest may occur. However, in the more arid southern slopes and the Pamir Region, forest cover is generally absent, transitioning directly to alpine meadows. The alpine nival zone, above 4,000–4,500 m, is permanently covered by snow and glaciers, where higher vascular plants are essentially nonexistent. Due to its overall high elevation, the Pamir Plateau features mainly alpine desert and steppe vegetation, which appears notably stunted and sparse.

Magnusson (1940) reported 16 *Lecanora* species from Qinghai, Gansu, and Xinjiang Provinces of China; among them, five *Lecanora dispersa* group species (*Lecanora caesioalutacea*, *L. hagenii*, *L. invadens*, *L. percrenata*, and *L. semipallida*) are currently named *Myriolecis*. This was the first comprehensive information on the *Lecanora* species in this study area. After half a century, Abbas and Wu (1998) re-illustrated two species (*Lecanora dispersa*, *L. hagenii*). To date, 16 species of *Myriolecis* have been reported in China (Magnusson 1940; Cao et al. 1995; Abbas et al. 2001; Mamut et al. 2019; Wei 2020; Payzulla and Mamut 2023, 2025).

To provide a taxonomic treatment of the species historically placed in the lichen genus *Myriolecis* in the arid zones of northwestern China, over 700 specimens were studied in the present study. An integrative taxonomic approach combining morphological-anatomical analysis, chemical analysis, and molecular biology was employed to classify the lichen specimens. The research results revealed three species new to science and three species newly recorded in China. Detailed morphological descriptions, illustrations, distribution data, and chemical information are provided for each species, as well as comparisons with morphologically similar and phylogenetically related species within the genus, information on the ecological environment of the study area, and a dichotomous key to all Chinese *Myriolecis* species.

## Materials and methods

### Taxon sampling and morphological, anatomical, and chemical studies

Field surveys for lichen diversity in northwestern China were carried out between 2003 and 2022, and from a total of 10 collection sites in Xinjiang, more than 700 specimens of *Myriolecis* were collected and stored in the “Lichen Research Center in Arid Zones of Northwest China.” Observations and measurements of thalli and apothecial characteristics of hand-cut sections, mounted in distilled water or 10% potassium hydroxide (KOH) (K), were studied using a Shunyu stereomicroscope SZM45-ST1, Motic-168 stereomicroscope, automatic biomicroscope (Ni-E), and standard light microscope (Nikon Eclipse E100). The granules were visible in polarized light (pol), with the following meanings: pol+, and pol-. The structure and coherency of paraphyses were also studied in K. In addition, the solubility of granules or microcrystals in the cortex and epithecium (pol+) was evaluated in 25% KOH (K) (Tripp et al. 2019) and 65% nitric acid (N) (Śliwa 2007). Both young and mature apothecia were examined to account for significant morphological changes in the apothecial margin and disc during development. Spot reactions of the thallus and apothecia with KOH (K), paraphenylenediamine [ethanol absolute] (PD), and sodium hypochlorite (C) were noted. Chemistry was studied with thin-layer chromatography (TLC) using solvent systems C and G (White and James 1985; Huneck and Yoshimura 1996; Orange et al. 2001). TLC analyses were undertaken on representative specimens of all accepted taxa.

### DNA extraction, PCR amplification, and sequencing

Total DNA was extracted with an Ezup Column Fungi Genomic DNA Purification Kit and Sangon Biotech Fungi Genomic DNA Extraction Kit in line with the manufacturers’ instructions. In addition, the modified CTAB method was used as the extraction procedure (Rogers and Bendich 1988). PCR amplification was undertaken for the internal transcribed spacer regions (ITS1 and ITS4) (Gardes and Bruns 1993; Larena et al. 1999) and the mitochondrial small subunit (mrSSU1 and mrSSU3R) (Zoller et al. 1999). The genomic DNA was amplified using polymerase chain reaction (PCR) in a 25 µL reaction mixture containing 2 µL of template DNA, 0.5 µL of each primer, 12.5 µL of MyTaq Mix, and 9.5 µL of double-distilled water. PCR amplifications for ITS were set up under the following conditions: 94 °C for 4 min (denaturation), 35 cycles of 94 °C for 30 s, 55 °C for 30 s (annealing), 72 °C for 60 s, and a final extension at 72 °C for 10 min, after which the samples were kept at 4 °C. mtSSU used a program of 95 °C for 10 min, followed by 35 cycles of 95 °C for 45 s, 50 °C for 45 s, and 72 °C for 90 s, with a final 10 min at 72 °C. The PCR product was sequenced by Sangon Biotech Co. Ltd. (Shanghai, China).

### Phylogenetic analysis

Forward and reverse sequences were checked and assembled using SeqMan v. 7.0.0 (DNASTAR); sequence alignments for each locus were performed with MUSCLE v. 5.0.1.102 (Edgar 2004) and improved manually using MEGA version 7 (Kumar et al. 2016) and MEGA version 5 (Tamura et al. 2011), respectively; and ambiguous areas were removed from the alignment by Gblocks (PhyloSuite v. 1.2.2) (Castresana 2000). PhyloSuite v. 1.2.2 was used to concatenate the aligned sequences of the different loci. The phylogenetic relationships of taxa were inferred using ML analyses conducted using IQ-TREE (Nguyen et al. 2015) in PhyloSuite v. 1.2.2. The best-fit substitution model was automatically selected by ModelFinder according to the Akaike information criterion (AIC), and the bootstrap values were calculated with 1000 pseudoreplicates, automatically selected as the best model for GTR+F+R5. Phylogenetic trees were visualized using FigTree v. 1.4.2 and the online editing software iTOL (https://itol.embl.de/) and analyzed and processed using Adobe Illustrator CS5 and Adobe Photoshop CC 2020.

## Results

### Phylogeny

All new sequences and reference sequences downloaded from GenBank for two gene loci were included in the alignments. The combined nrITS + mtSSU dataset included 153 operational taxonomic units (OTUs) (100 for ITS and 53 for SSU) representing 57 taxa, and the outgroup is provided in the supplementary materials (Suppl. material 1), among which 39 new sequences (20 for ITS and 19 for SSU) were generated in this study and deposited in GenBank (https://www.ncbi.nlm.nih.gov/genbank/). Seven species of *Ramboldia* were chosen as the outgroup. The related sequences were obtained from a BLAST search and from recently published data, and the combined gene analyses share similar overall topologies at the generic level and are in agreement with previous studies (Zhao et al. 2016; Ivanovich et al. 2021).

**Figure 1.**
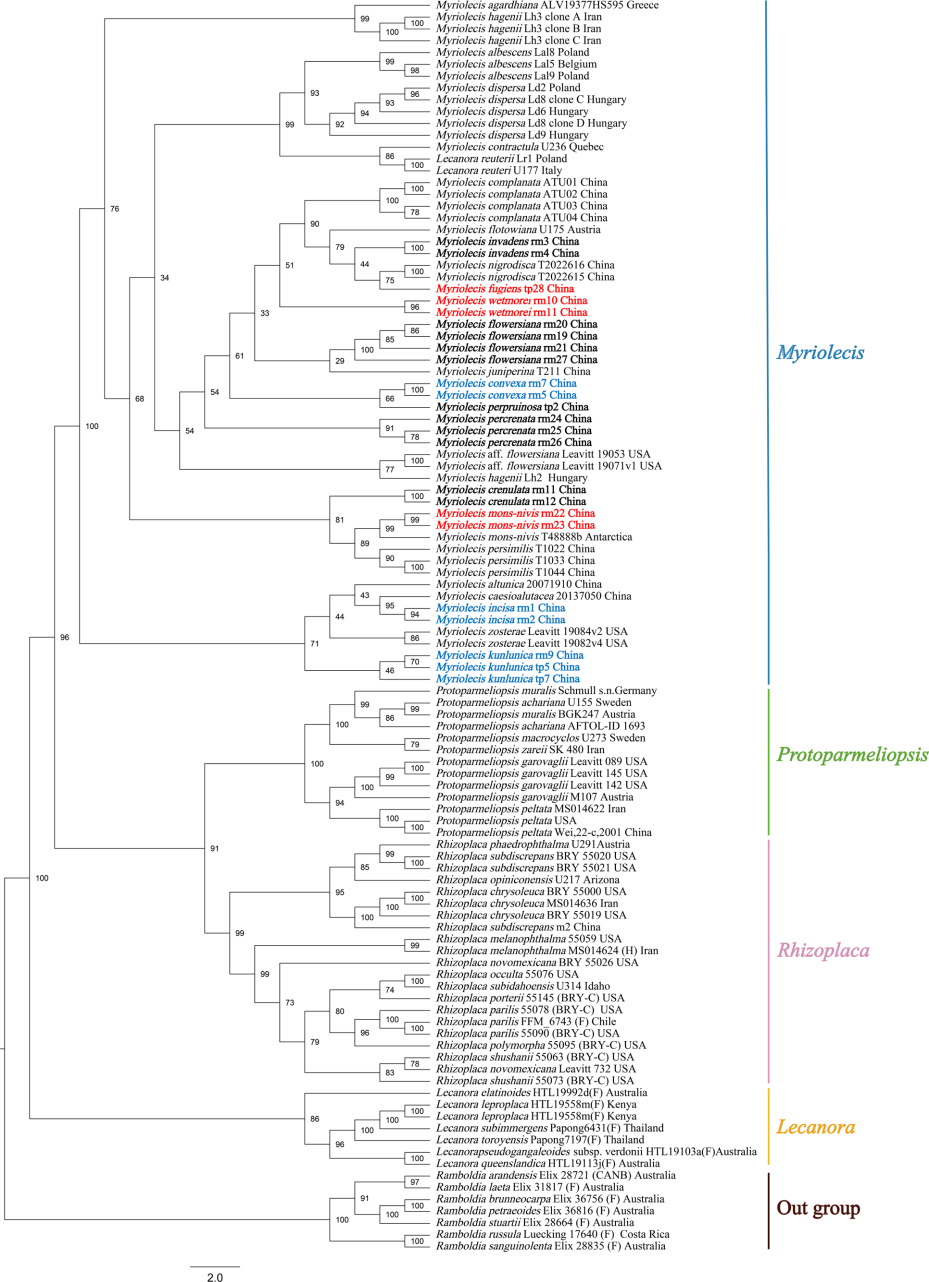
The IQ-TREE maximum likelihood tree of *Myriolecis* species, based on the concatenated ITS + mtSSU dataset. The numbers in each node represent bootstrap support (BS) values. Names of novel taxa (blue), new records (red) described in this paper, and related *Myriolecis* species extracted for this research are in bold.

The resulting ML phylogenetic tree is shown in Fig. 1. In the present phylogenetic tree, all *Myriolecis* species formed a monophyletic lineage, which, together with *Protoparmeliopsis*, constituted a strongly supported sister clade. The new species (*M. convexa*, *M. incisa*, and *M. kunlunica*) and new records (*M. fugiens*, *M. mons-nivis*, and *M. wetmorei*) were resolved into the section *Myriolecis*. In the phylogenetic tree, some branches within the *Myriolecis* clade exhibit relatively low bootstrap support values. This phenomenon highlights the need for incorporating more gene fragments or increasing taxon sampling in future similar studies.

### Taxonomy

#### New species

##### Myriolecis convexa

Taxon classificationFungiLecanoralesLecanoraceae

R.Mamut sp. nov.

2C7EC977-E1B5-5EB3-BA3B-96E08B3C4074

855085

Fig. 2

###### Etymology.

The epithet “convexa” refers to the convex shape of the apothecia.

**Figure 2.**
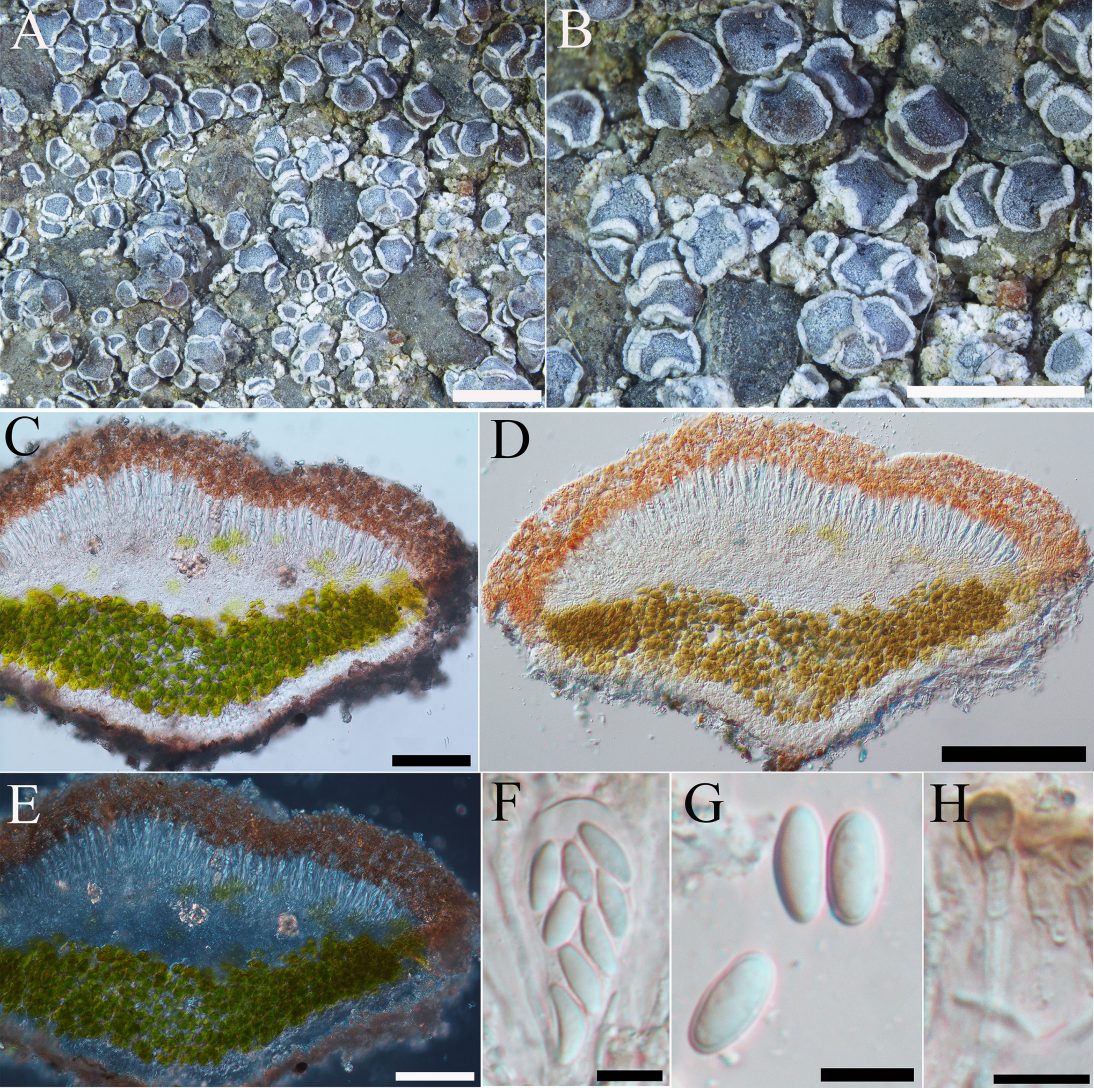
*Myriolecis convexa* (XJU20108046, holotype). **A, B**. Morphological structure of thallus and apothecia; **C**. Apothecial anatomy in regular light; **D**. Section of apothecium in regular light after application of N; **E**. Section of apothecium in polarized light; **F**. Ascus; **G**. Ascospores; **H**. Paraphysis. Scale bars: 2 mm (**A, B**); 200 μm (**C, E**); 100 μm (**D**); 10 μm (**F–H**).

###### Diagnosis.

The diagnostic characteristics of the new species have dispersed warts on the thallus and often convex apothecial disc, epithecium shades of reddish-brown, distinctly granular, granules insoluble in N and K and turn orange in N.

###### Description.

Thallus clearly visible, crustose, small dispersed warts or fine granulose or present only under apothecia, shades of pale gray or whitish-gray to white; prothallus not seen; Apothecia numerous, usually often clustered in groups of 2–3 apothecia, sessile to slightly constricted at base, 0.4–1.2 mm; Disc reddish-brown to brown or chocolate brown, usually heavily white pruinose, plane or, when apothecia crowded, then more irregular and the disc becoming.

Considerably convex; margin prominent, smooth, young ones regular in shape, round, older ones usually slightly flexuose, white to whitish-gray or becoming same color as the thallus, slightly pruinose, raised above or level with disc or, when disc strongly convex margin, not very clearly visible; Amphithecium present, distinctly divided into algal layer and cortex, algae occur as algal layer under hypothecium, discontinuous; cortex thin, distinctly expanded at the base, with granules (pol ± insoluble in K and soluble in N); Epithecium brown, reddish-brown, not at all granular (pol ±, insoluble in K and N), N+ turning orange; Hymenium hyaline, 40–95 μm high; Subhymenium distinct, hyaline, to 35–45 μm high; Hypothecium hyaline, composed of gelatinized hyphae, hyphae more clear in K and N, 50–130 μm high; Paraphyses simple or slightly branched at tips, thickened, slightly expanded apically, often brown pigmented at tips, free in K. Asci clavate, 8-spored; Ascospores hyaline, simple, ellipsoid to narrowly ellipsoid, 6.5–15 × 4.5–6.5 μm. Pycnidia not seen.

###### Chemistry.

Apothecial margin K–, C–, KC–, PD–; disc K–, C–, KC–, PD–; No lichen products detected by TLC.

###### Habitat.

on rock.

###### Notes.

*M. convexa*, in many aspects, shares characteristics similar to those of other species of the genus *Myriolecis*, such as scattered to crowded apothecia with a mostly white thalline margin, narrowly to broadly ellipsoid ascospores, and no lichen products. However, *M. convexa* differs from all species by its small, dispersed, areolate thallus and usually distinctly convex apothecial discs. Morphologically, *M. convexa* is similar to *M. hagenii* (Ach.) Śliwa, Zhao Xin & Lumbsch in having heavily pruinose, reddish-brown apothecia; however, *M. hagenii* never has a distinct thallus, and its epithecium usually has K-soluble granules and smaller apothecia with a much thinner, entire to incised apothecial margin (Śliwa and Hawksworth 2006; Śliwa 2007).

###### Type species.

**China** • Xinjiang, Urumqi city, middle part of Tianshan Mountains, 43°22'43"N, 86°47'50"E, 1864 m a.s.l., on rock, 15 Aug 2010, *R. Mamut* (***holotype*** designated here 20108046).

###### Additional specimens examined.

**China** • Xinjiang, Urumqi City, Bayi Forest Farm, 1710 m a.s.l., 43°22'48.1"N, 86°48'40.2"E, on rock, 28 Aug 2007, R. Mamut 20071962-a, 20071962-b (XJU); • 1864 m a.s.l., 43°22'43"N, 86°47'50"E, on rock, 15 Aug 2010, R. Mamut 20108078, 20108079, 20108081, 20108082, 20108083(XJU); • 2800 m a.s.l., 43°44'61"N, 86°48'719"E, on rock, 10 May 2010, R. Mamut 20108047(XJU); • 2270 m a.s.l., 43°21'27"N, 86°47'32"E, on rock, 29 May 2021, R. Mamut 20210371(XJU); • 1780 m a.s.l., 43°23'33.5"N, 86°47'71"E, on rock, 15 Jul 2022, R. Mamut T20221204(XJU); • Xinjiang, Urumqi City, Hou Xia, 2060 m a.s.l., 43°00'00"N, 86°34'375"E, on rock, 6 Oct 2009, R. Mamut 20090106(XJU); • Xinjiang, Changji, Ha Xiong Gu, 1870 m a.s.l., 43°48'626"N, 87°59'658"E, on rock, 8 Jul 2013, R. Mamut 20130708(XJU).

##### Myriolecis incisa

Taxon classificationFungiLecanoralesLecanoraceae

R.Mamut sp. nov.

0FBB9E55-7BE6-5E5C-9B8B-C31F38C3F16D

861339

Fig. 3

###### Etymology.

The epithet “incisa” refers to the incised apothecial margin of this new species.

**Figure 3.**
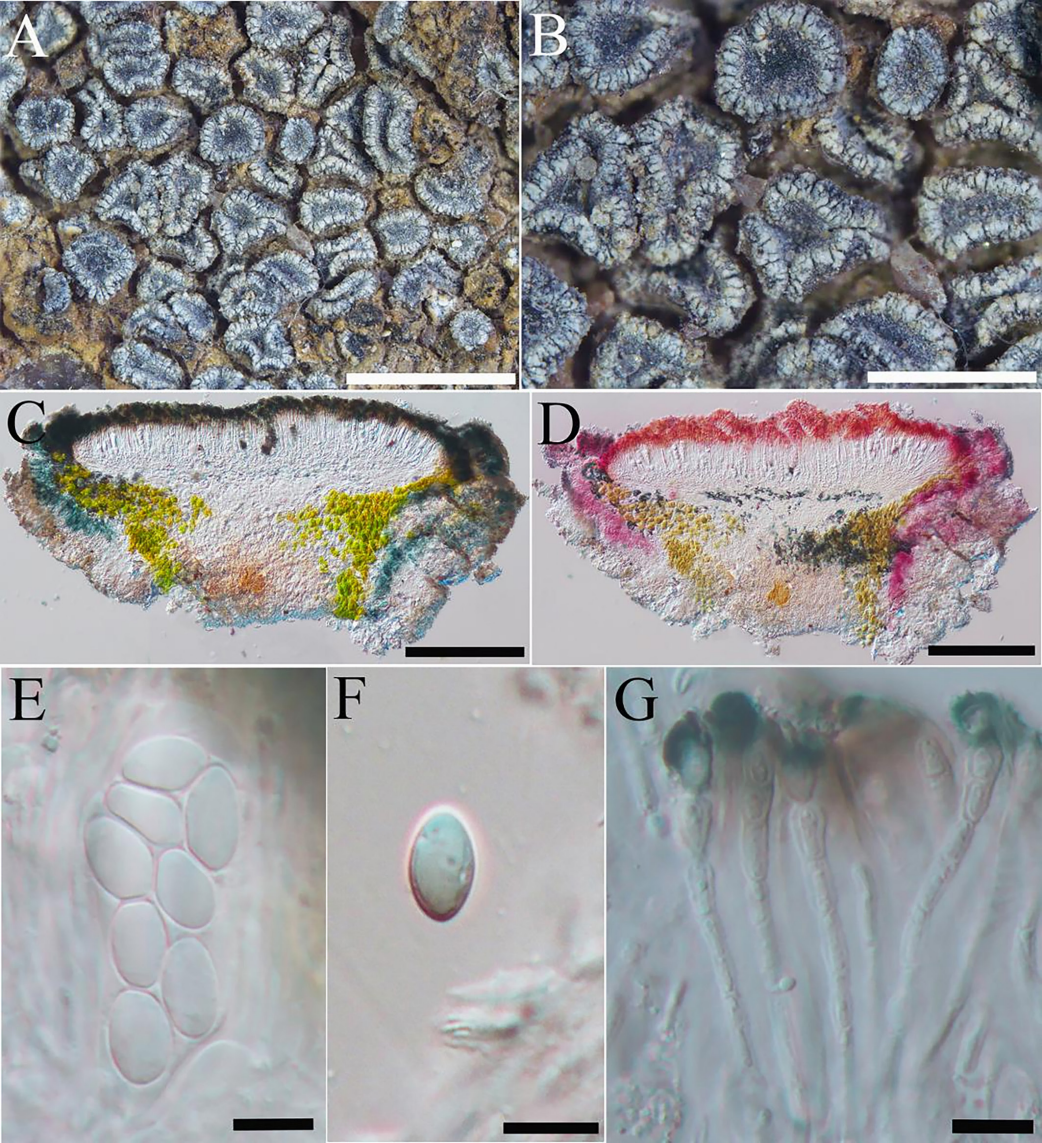
*Myriolecis incisa* (XJU20080999, holotype). **A, B**. Morphological structure of thallus and apothecia; **C**. Apothecial anatomy in regular light; **D**. Section of apothecium in regular light after application of N; **E**. Ascus; **F**. Ascospores; **G**. Paraphyses. Scale bars: 2 mm (**A, B**); 100 µm (**C, D**); 10 µm (**E–G**).

###### Diagnosis.

The new species has whole cortex with bluish-green pigment (this part becoming pink in N) and epithecium shades of olive to dark brown (turning red in N). *M. incisa* is also well recognized by the small crenated apothecial margin.

###### Description.

Thallus crustose, moderately thick (0.1–0.3 mm), areolate to rimose, but not leprose, margin determinate, not or rarely pruinose, shades of brown to yellowish-brown or dark gray; Prothallus not visible. Apothecia usually numerous, lecanorine, sessile, densely covering most of thallus, emerging on the surface of thallus, 0.3–1.0 mm diameter, occurring singly; disc plane, almost black, epruinose to slightly pruinose; Thalline margin prominent, raised above discs, rough, incised to distinctly crenate, epruinose, most often white; Amphithecium present, with a discontinuous algal layer, algae sparse; cortex distinctly delimited, clearly expanded at the base, 45–60 μm wide laterally and 25–35 μm wide at the base, often obviously bluish pigmented and more intense and becoming pink in N, not granulose; Epithecium olive to dark brown, distinctly granular, granules superficial and between paraphyses tips (pol ±), slowly disappearing in K and also often turning red in N; Hymenium hyaline, 45–70 μm high; Subhymenium indistinct to distinct, hyaline, thin, 15–20 μm high; Hypothecium hyaline, 40–110 μm high, composed of gelatinized hyphae; Paraphyses slender, simple, thick, slightly expanded, distinctly septate, deep green pigmented at the tips, coherent in K. Asci clavate, 8-spored; Ascospores hyaline, simple, ellipsoid, 7–13 × 4.5–8.5 μm. Pycnidia not seen.

###### Chemistry.

Apothecial margin K–, C–, KC–, PD–; disc K–, C–, KC–, PD–; No lichen products detected by TLC.

###### Habitat.

On calcareous rock.

###### Notes.

This species is easily recognizable due to its distinctly developed rimose-areolate thallus and its almost black, either epruinose or only slightly pruinose, apothecial disc with an incised margin. Additionally, the species carries a bluish-green pigment covering the entire cortex, along with shades of olive to dark brown in the epithecium that turns pink and red in N. These characteristics are not observed in other species of the same genus. *Myriolecis incisa* is similar to *M. perpruinosa* (Fröberg) Śliwa, Zhao Xin & Lumbsch and *M. caesioalutacea* (H.Magn.) R.Mamut.

However, *M. perpruinosa* usually has submoniliform paraphyses, an ash-gray thallus, and bluish-white pruina on the dark apothecial disc. In addition, *M. perpruinosa* has greenish cortex cell walls, with only the outer part of the cortex N+ purple (Fröberg 1997; Śliwa 2007; Muchnik and Śliwa 2011). *M. caesioalutacea* is characterized by a bluish-white pruinose apothecial disc, a thallus-colored apothecial margin (ashy brown, brown to dark brown), and a thallus containing 2,7-dichlorolichexanthone (Magnusson 1940; Mamut et al. 2019). The new species is morphologically similar to *M. hagenii* in its epruinose to slightly whitish pruinose apothecial disc, which has an incised rather than distinctly crenate apothecial margin. However, *M. hagenii* differs from the new species by having small apothecia up to 0.8 mm diam., with an orange to reddish tint and a maroon to brown disc. *M. hagenii* also has a considerably thinner amphithecial cortex (Śliwa 2007; De la Rosa et al. 2012).

###### Type species.

**China** • Xinjiang, Kashkar, Kargilik County, Mt. Kunlun, 36°34'92"N, 77°00'36"E, 2880 m a.s.l, on rock, 30 Jun 2008, *R. Mamut* (***holotype*** designated here 20080999).

###### Additional specimens examined.

**China** • Kashkar, Kargilik County, Mt. Kunlun, 2880 m a.s.l, 36°34'92"N, 77°00'36"E, on rock, 30 Jun 2008, R. Mamut 20080997, R. Mamut 20080998, R. Mamut 20080994, R. Mamut 20080995, R. Mamut 20080996, R. Mamut 20080986, R. Mamut 20080961 (XJU).

###### Ecology and distribution.

*M. incisa* is known only from the Kunlun Mountains. It grows on calciferous rock, often with other members of *Myriolecis*, for example, *M. altunica* R. Mamut and *M. caesioalutacea* and overgrowing or commensally on other lichens, for example, *Caloplaca* spp.

##### Myriolecis kunlunica

Taxon classificationFungiLecanoralesLecanoraceae

R.Mamut sp. nov.

029AD596-BA6D-58B5-9C09-2E8B4FE74760

861340

Fig. 4

###### Etymology.

“kunlunica” according to the specimen collection location, Kunlun Mountain.

**Figure 4.**
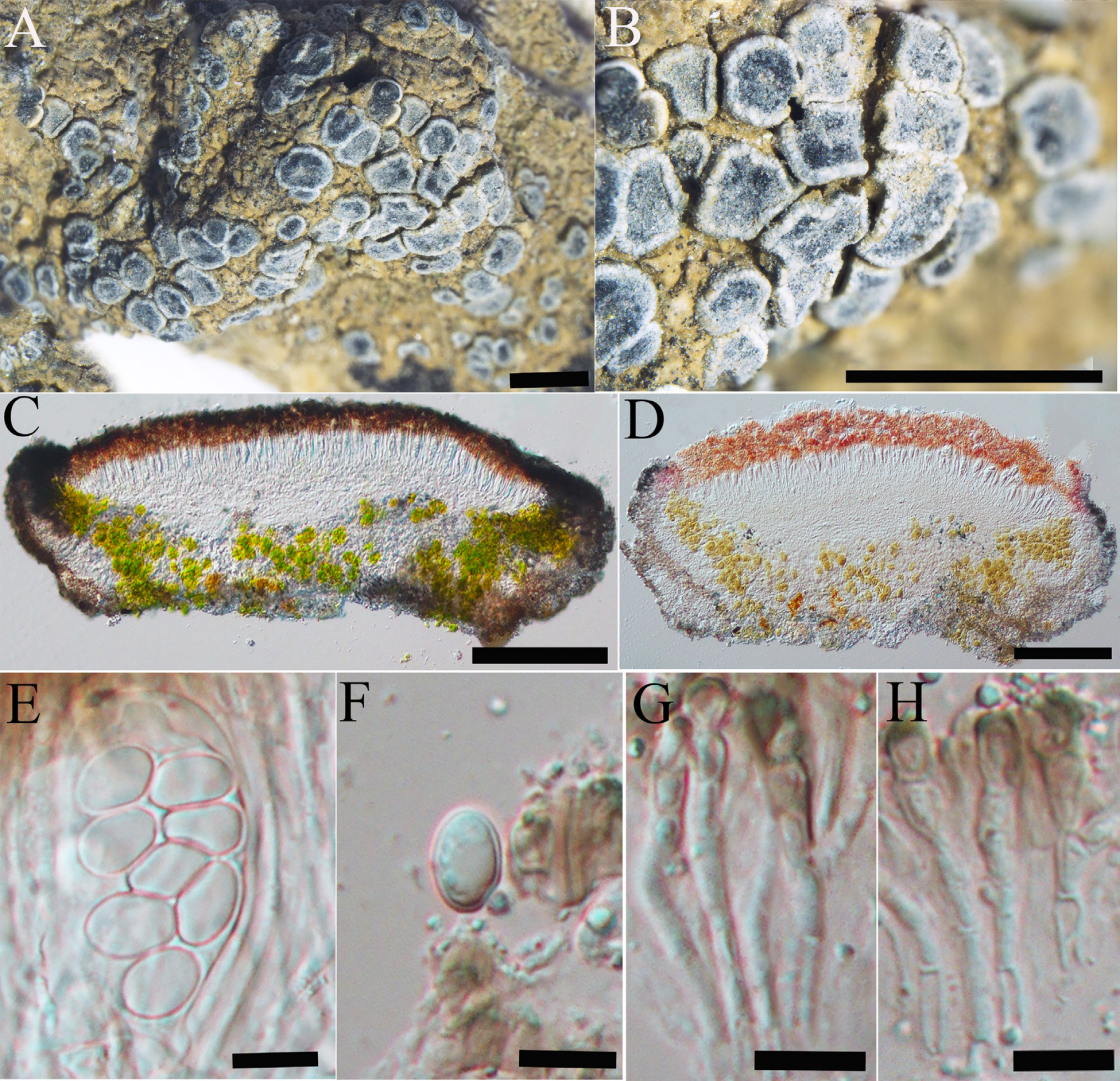
*Myriolecis kunlunica* (XJU20090729, holotype). **A, B**. Morphological structure of thallus and apothecia; **C**. Apothecial anatomy in regular light; **D**. Section of apothecium in regular light after application of N; **E**. Ascus; **F**. Ascospores; **G, H**. Paraphyses. Scale bars: 2 mm (**A, B**); 200 µm (**C, D**); 10 µm (**E–H**).

###### Diagnosis.

*Myriolecis kunlunica* is characterised by its clearly visible, often forming distinct rimose thallus and the white heavily pruina on the dark apothecial disc and chemical characters (2, 7- dichlorolichexanthone).

###### Description.

Thallus crustose, usually distinct, continuous, forming areolate to rimose, sometimes partly superficial, shade of brown to dark brown; Prothallus not visible; Apothecia abundant, lecanorine, occurring scattered to crowded, sessile, 0.5–1.2 mm diam.; Disc plane, almost black, heavily pruinose, never epruinose; Thalline margin prominent, thick, smooth, level with the disc or raised above discs, slightly flexuose, when old, apothecia white, pruinose; Amphithecium with algae and cortex, algal layer discontinuous, algae not very dense; Cortex well delimited, but not very thick, slightly expanded at the base, strongly expanding in K and N, N+ pink upper part, with granules (well-visible in polarised light, insoluble in K, soluble in N); Epithecium brown to red brown or olive, unchanged with K, but N+ reddish or deep orange, not at all granular; Hymenium hyaline, 35–70 μm high; Subhymenium distinct, hyaline, thin, 25–55 μm high; Hypothecium hyaline, 40–110 μm high, composed of gelatinized hyphae; Paraphyses slender, simple, expanded apically, distinctly septate, brown pigmented, coherent in K. Asci clavate, 8-spored; Ascospores hyaline, simple, ellipsoid (broadly ellipsoid to ellipsoid), 7–12 × 4.5–7.5 μm. Pycnidia not seen.

###### Chemistry.

Apothecial margin K–, C–, KC–, PD–; disc K–, C–, KC–, PD–; 2,7-dichlorolichexanthone detected by TLC.

###### Habitat.

On calcareous rock.

###### Notes.

*M. kunlunica* is characterized by its much more abundant thallus, usually pruinose and almost black apothecial discs, the presence of *dispersa*-like granules in the epithecium (insoluble in K and N, N+ red), and a black-green color at the very top of the margin (N+ purple). *M. kunlunica* is similar to *M. perpruinosa*, but the latter differs in having an ash-gray thallus and bluish-white pruina, a black or dark brown apothecial disc, and thick, submoniliform paraphyses.

###### Type species.

China • Xinjiang, Kashkar, Kargilik County, Mt. Kunlun, 36°58'33"N, 75°32'18"E, 4055 m a.s.l., on rock, 29 Jul 2008, *R. Mamut* (***holotype*** designated here 20090729).

###### Additional materials examined.

China • Xinjiang, Kashkar, Kargilik County, Mt. Kunlun, Serik Daban, 36°58' 33"N, 75°32'18"E, 4055 m a.s.l., on rock, 29 Jul 2008, R. Mamut 20040671, 200801001, 20081009, 20080989 (XJU).

###### Ecology and distribution.

*M. kunlunica* is known only from the Kunlun Mountains. It grows on calciferous rock, often with *Aspicilia* sp. and other members of *Myriolecis*.

#### New record species

##### Myriolecis fugiens

Taxon classificationFungiLecanoralesLecanoraceae

(Nyl.) Śliwa, Zhao Xin & Lumbsch.

368F5966-0948-5391-948F-9898C7D31633

Fig. 5

###### Description.

Thallus not well developed or only evident around the apothecia; prothallus not seen; Apothecia rounded, usually not common, occurring singly, sessile, when apothecia constricted at the base, apothecia distinctly cup-shaped; Disc apparently pruinose, yellowish-brown to pale reddish-brown, sometimes also pale yellow, flat; thalline margin present, thick, level with the disc or ± higher, pruinose, white; Amphithecium indistinct to distinctly divided into cortex and algal layer, algae dense or sparse; cortex expanded at the base, granular (pol +, insoluble in K, soluble in N); Epithecium brown to reddish-brown, granular (pol ±, soluble in K and insoluble in N); Hymenium hyaline, to 50–75 μm high; Subhymenium indistinct; Hypothecium hyaline, 95–130 μm high, composed of gelatinized hyphae, more clear in K. Paraphyses slightly branched, slightly or not expanded, without swelling or pigment cap in apical cell, free in K. Asci clavate, 8-spored; Ascospores hyaline, simple, mostly ellipsoid (broadly ellipsoid to ellipsoid), 8.5–12 × 4.5–6 μm; Pycnidia not seen.

**Figure 5.**
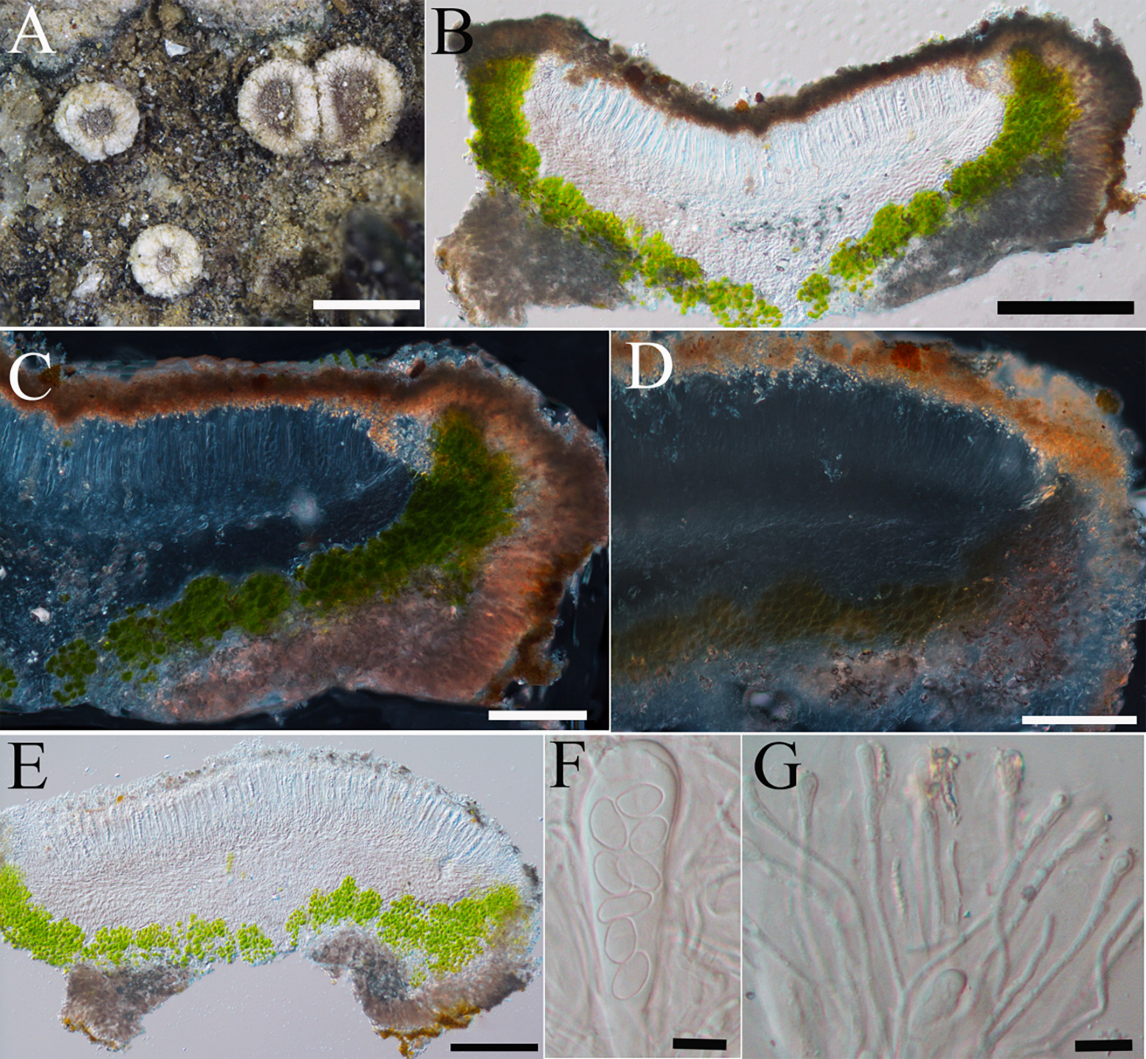
*Myriolecis fugiens* (Herb. No. 20136167). **A**. Morphological structure of thallus and apothecia; **B**. Apothecial anatomy in regular light; **C**. Section of apothecium in polarized light; **D**. Section of apothecium in polarized light after application of N; **E**. Section of apothecium in regular light after application of K; **F**. Ascus; **G**. Paraphysis. Scale bars: 1 mm (**A**); 200 μm (**B**); 100 μm (**C, D**); 200 μm (**E**); 10 μm (**F, G**).

###### Habitat.

On calcareous rock.

###### Chemistry.

Apothecial margin K+ yellow, C+ orange, KC+ yellow to orange, PD+ pale orange; disc K+ yellow, C+ orange, KC+ orange, PD+ orange. Pannarin detected by TLC.

###### Notes.

*M. fugiens* was first described by James in 1960 (James 1960), who provided a detailed morphological description. Morphologically, *M. fugiens* is similar to *M. invadens* (H.Magn.) Śliwa, Zhao Xin & Lumbsch and *Myriolecis flowersiana* (H.Magn.) Śliwa, Zhao Xin & Lumbsch. However, *M. fugiens* can be distinguished from these species by its chemistry, specifically the consistent presence of pannarin, as well as its spot test reactions: C+ orange, KC+ orange, and PD+ orange. Additionally, *M. fugiens* differs in its anatomy, notably by having K-soluble granules in the epithecium and N-soluble granules in the cortex.

###### Specimens examined.

**China** • Xinjiang, Atux, Aketao County, Oytag, Mt. Southern Tianshan, 38°53'54"N, 75°11'57"E, 2930 m a.s.l., on rock, 27 Aug 2013, *R. Mamut* 20136188-a, 20136188-b, 20137179-1 (XJU).

##### Myriolecis mons-nivis

Taxon classificationFungiLecanoralesLecanoraceae

(Darb.) Śliwa, Zhao Xin & Lumbsch.

43A16D87-4BDC-5FB8-AAC4-CACE7F349525

Fig. 6

###### Description.

Thallus clearly visible, thin, continuous, more or less rimose, edge indefinite, brown to dark brown; prothallus not seen; Apothecia numerous, usually clustered in groups or occurring singly, sessile or constricted at the base, 0.3–0.8 mm in diameter; Disc plane smooth, dark brown to dark blue, epruinose; thalline margin level with the disc, rough, cracked with numerous small fissures or flexuose or incised, epruinose, white to grayish-white; Amphithecium not well delimited into algal layer and cortex, algal layer with algae abundant filling, continuous; cortex indistinctly divided, composed of granules (pol +, insoluble in K and N); Epithecium shades of reddish-brown to brown, granules superficial and inspersed in paraphyses tips (pol ±, insoluble in K and N); Hymenium hyaline, 45–65 μm high; Subhymenium indistinct or distinct, thin, hyaline; Hypothecium hyaline 40–65 μm high, composed of gelatinized hyphae (most clear in K); Paraphyses slender, simple, never branched, expanded to capitate apically, red-brownish pigmented, with sharply delimited as a cap (up to 0.3 μm), coherent in K. Asci clavate, 8-spored; Ascospores hyaline, simple, broadly ellipsoid to narrowly ellipsoid, 7–12 × 5–8 μm, Pycnidia not seen.

**Figure 6.**
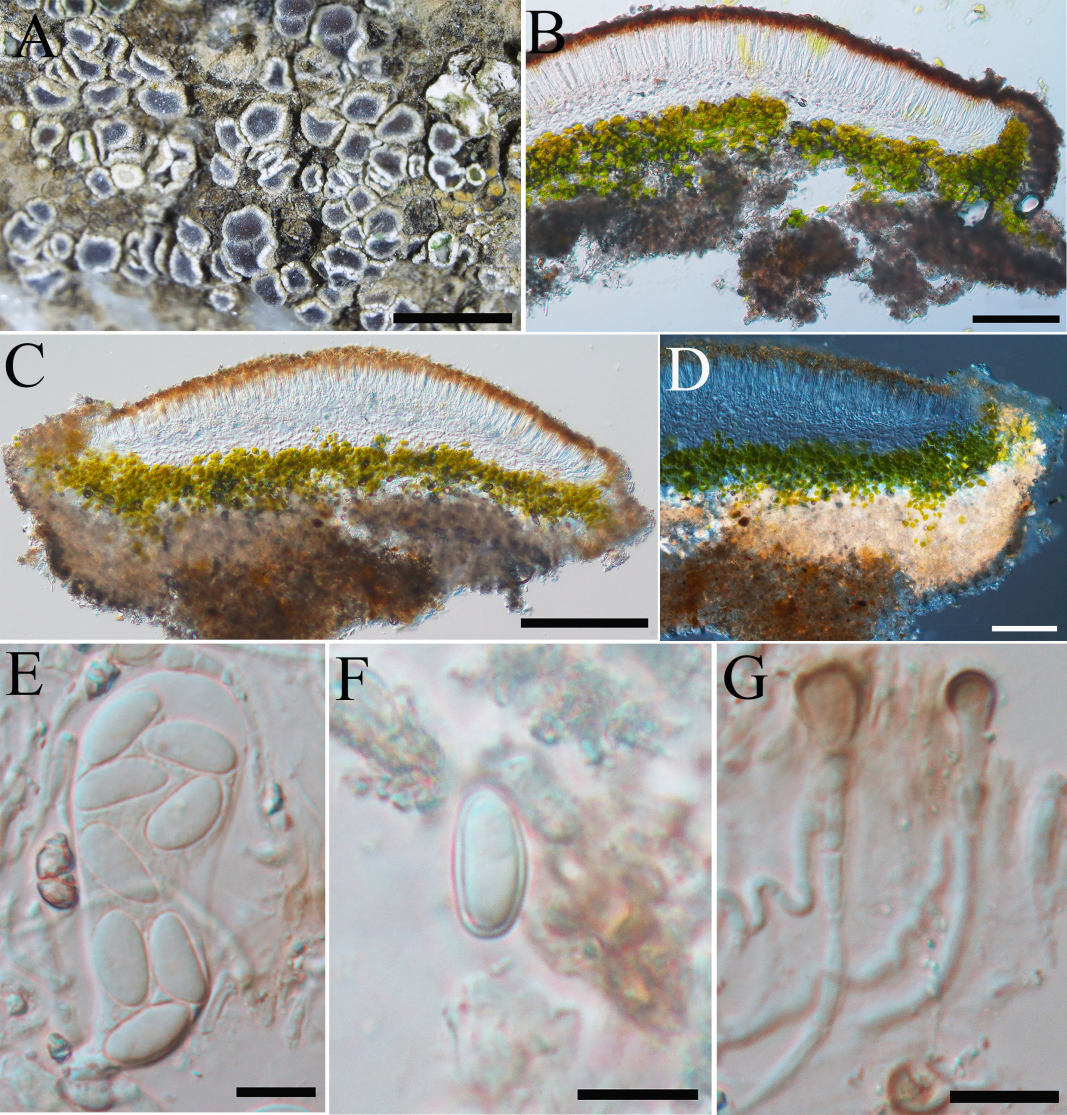
*Myriolecis mons-nivis* (Herb. No. 201367). **A**. Morphological structure of thallus and apothecia; **B**. Apothecial anatomy in regular light; **C**. Section of apothecium in regular light after application of K; **D**. Section of apothecium in polarized light after application of K; **E**. Ascus; **F**. Ascospores; **G**. Paraphyses. Scale bars: 2 mm (**A**); 100 µm (**B–D**); 10 µm (**E–G**).

###### Chemistry.

Apothecial margin K –, C –, KC –, PD –; disc K–, C–, KC–, PD–; No lichen products detected by TLC.

###### Habitat.

On calcareous rock.

###### Notes.

The species can be easily identified by its clearly visible, rimose thallus and dark brown, usually epruinose apothecial disc, as well as by its white to ash-gray apothecial margin with slight cracks and reddish-pigmented epithecium. This species is closely related to *M. flowersiana*, which differs in its apothecial color and has more submoniliform, pigmented, and distinctly septate paraphyses as well as narrowly ellipsoid spores. Additionally, the epithecium of *M. flowersiana* is deeply pigmented, with up to one-third of the upper hymenium exhibiting more intense red coloration (Śliwa 2007; De la Rosa et al. 2012). Morphologically, the newly recorded species is identical to *M. dispersa* (Pers.) Śliwa, Zhao Xin & Lumbsch; however, *M. dispersa* has an inconspicuous thallus and a distinctly granular epithecium that is tinged yellow or brown, contains pannarin, and has a PD+ orange reaction of the apothecial margin (Fröberg 1997; Śliwa 2007; Muchnik and Śliwa 2011; De la Rosa et al. 2012).

###### Specimens examined.

**China** • Xinjiang, Atux, Aketao County, Oytag, Mt. Southern Tianshan, 38°53'54"N, 75°11'57"E, 2930 m a.s.l., on rock, 27 Aug 2013, *R. Mamut* 201367, 201368, 201366, 201369, 201364, 201363(XJU).

##### Myriolecis wetmorei

Taxon classificationFungiLecanoralesLecanoraceae

(Śliwa) Śliwa, Zhao Xin & Lumbsch.

7D072966-13D6-57F2-BF07-8592D59AEE90

Fig. 7

###### Description.

Thallus not clearly visible within the substratum and present only under apothecia or poorly developed; prothallus not seen; Apothecia lecanorine, sessile, constricted at the base, occurring singly and usually rarely clustered in groups, 0.4–1.0 mm diam.; disc black, strongly pruinose, never epruinose, smooth, flat; thalline margin prominent, level with the disc, smooth, thick, heavily white pruinose; Amphithecium developed with distinctly delimited into algal layer and cortex, algae abundant and dense, continuous; cortex strongly expanded at the base, 15–35 μm wide laterally and 50–65 μm wide at the base, composed of granules (pol +, insoluble in K, soluble in N); Epithecium brown to dark brown, N+ reddish at beginning and then soon disappearing, granules superficial (pol+, soluble in K, insoluble in N); Hymenium hyaline, 45–65 μm high; subhymenium distinct, 25–65 μm high; Hypothecium hyaline, 35–50 μm high, composed of gelatinized hyphae. Paraphyses simple, rarely branched, expanded, pigmented, free in K. Asci clavate, 8-spored; Ascospores hyaline, simple, ellipsoid (broadly ellipsoid to ellipsoid), 8.5–12 × 4.5–6.5 μm; Pycnidia not seen.

**Figure 7.**
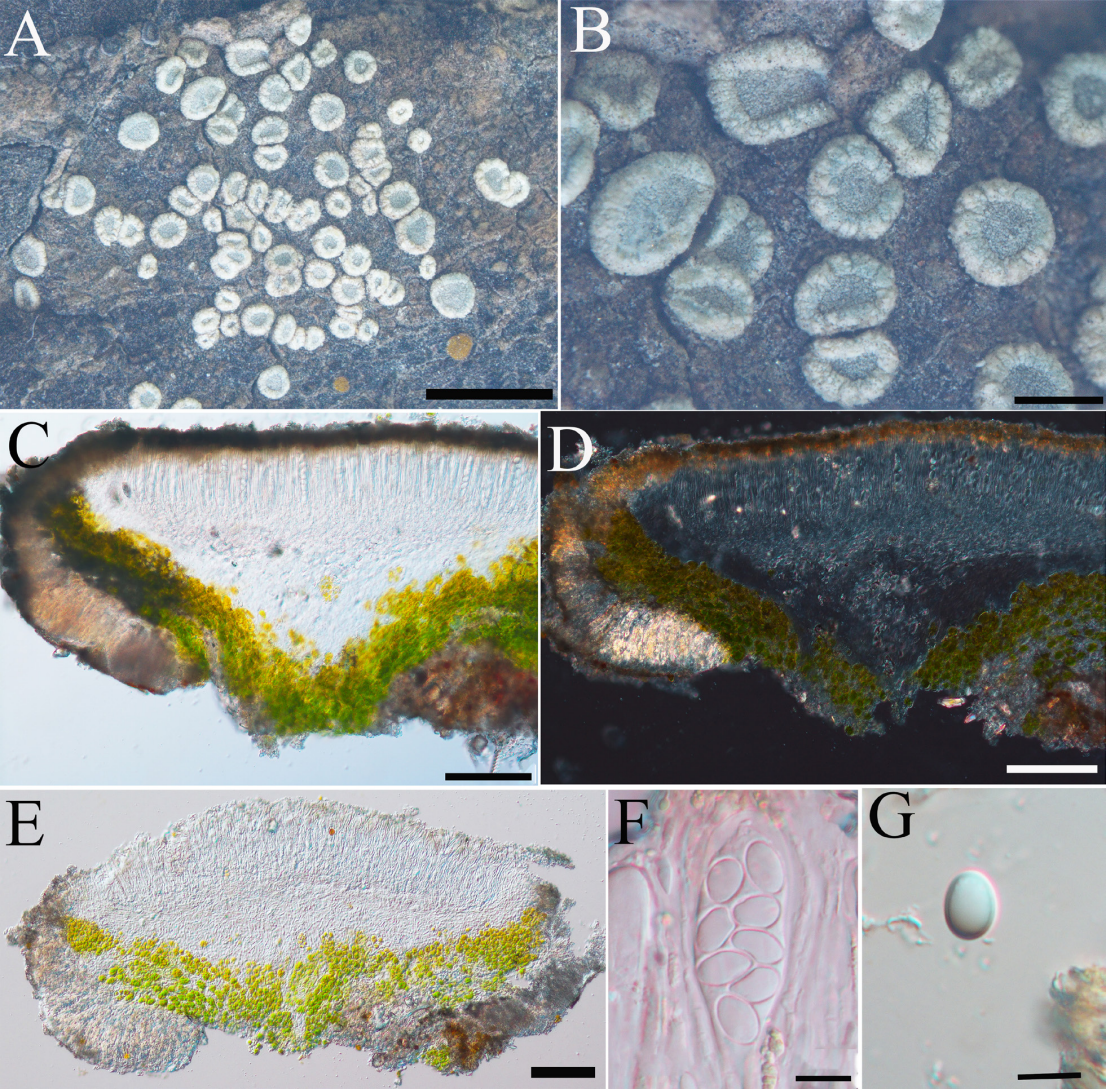
*Myriolecis wetmorei* (Herb. No. 20137264). **A, B**. Morphological structure of thallus and apothecia; **C**. Apothecial anatomy in regular light; **D**. Section of apothecium in polarized light; **E**. Section of apothecium in regular light after application of K; **F**. Ascus; **G**. Ascospores. Scale bars: 2 mm (**A**); 500 μm (**B**); 100 µm (**C–E**); 10 µm (**F, G**).

###### Chemistry.

Apothecial margin K–, C–, KC–, PD–; disc K–, C–, KC–, PD–; No lichen products detected by TLC.

###### Habitat.

Corticolous species occurring on tree bark.

###### Notes.

*M. wetmorei* is sometimes difficult to separate from *M. hagenii* and *M. perpruinosa*, as the three species possess finely pruinose apothecia. *M. hagenii* is distinguished on account of smaller apothecia (up to 0.8 mm diam.) with a much thinner, entire to incised rather than cracked apothecial margin. *M. perpruinosa* differs from *M. wetmorei* by having a clearly visible thallus and greenish cortex cell walls, with the outer part of the cortex turning purple in N (Fröberg 1997; Śliwa 2007; Muchnik and Śliwa 2011). Morphologically, the newly recorded species is identical to *M. torrida* (Vain.) Śliwa, Zhao Xin & Lumbsch, but the latter species is easily separable by its more warted and rimose-areolate thallus. In addition, anatomically, it differs in having its epithecium colored olive-green, intensely green with K, and turning reddish-brown or red in N (Śliwa and Olech 2002; Śliwa 2007).

###### Specimens examined.

**China** • Xinjiang, Urumqi City, Mt. Middle Tianshan, Yingxiong Bridge, 43°28'08"N, 87°05'48"E, 2110 m a.s.l., on bark, 15 Aug 2006, *R. Mamut* 060815 (XJU); • Aksu City, Kucha County, Mt. Southern Tianshan, Tilmat Daban, 2450 m a.s.l., on bark, 13 May 2007, *R. Mamut* 20121719, 2003111 (XJU); • Atux City, Aketao County, Oytag, Mt. Southern Tianshan, 2930 m a.s.l., 38°53'25"N, 75°11'57"E, on bark, 27 Aug 2013, *R. Mamut* 20137264 (XJU).

### Key to known species of *Myriolecis* in China

**Table:** 

1	Secondary metabolites present (TLC)	**2**
–	Secondary metabolites absent (TLC)	**8**
2	Thallus and apothecial margins K+ yellow, KC+ yellow, PD+ orange; contains pannarin or vinetorin	**3**
–	Thallus and apothecial margin K–, C–, KC–, PD–; contains 2, 7-dichlorolichexanthone	**4**
3	Contains pannarin	**5**
–	Contains vinetorin	**6**
4	Epithecium brown to black brown, pale bluish green in K, pale red in N	***M. caesioalutacea***
–	Epithecium brown to red brown, or olive, K-, but reddish or deep orange in N	***M. kunlunica***
5	Apothecia rare, 0.3–1.2 mm in diam., disc yellowish brown to pale reddish brown, epithecium granulose soluble in K	***M. fugiens***
–	Apothecia abundant, 0.3–0.9 mm in diam., disc color variable, brown to black brown, or to black; epithecium granulose insoluble in K	***M. dispersa***
6	Apothecial disc yellowish-gray, pale brown, yellowish to yellow-orange, epruinose or faintly yellowish pruinose	***M. semipallida***
–	Apothecial disc brown to dark brown, black or plumbeous, usually with slight to thick white pruina	**7**
7	Thallus well-developed, distinctly visible, thick, verrucose, rimose-verrucose, gray, grayish-white, chalky; apothecia 0.3–3.5 mm in diam.; disc black; epithecium brown or reddish-brown, N + reddish to orange	***M. gigantea***
–	Thallus crustose, or only attached to the base of apothecia or indistinct, greenish-brown to brown, containing bluish pigment; apothecia 0.2–1.0 mm in diam.; disc brown to dark brown or black, sometimes plumbeous, epithecium brown, dark brown or olive-brown, often green pigmented, N + faintly pink	***M. invadens***
8	Apothecial margin cracked	**9**
–	Apothecial margin entire to undulate or slightly crenate	**14**
9	Epithecium pigmented	**10**
–	Epithecium unpigmented	**13**
10	Paraphyses moniliform	**11**
–	Paraphyses usually pigmented, apices swollen	**12**
11	Apothecial disc reddish brown, dark brown to black; not pruinose; epithecium pigmented (pigment may extend to the upper third of the hymenium); ascospores generally oblong-ellipsoid, 13–17 × 4.5–6.5 μm	***M. flowersiana***
–	Apothecial disc brown, black or usually black; usually with slight pruina; epithecium brown or olive-green, or bluish green; ascospores ellipsoid, 8–16 × 4.5–7.5 μm	***M. percrenata***
12	Thallus well-developed, clearly visible, areolate; apothecial disc concave, margin irregular, rough, not pruinose; epithecium olivaceous, greenish-black, or greenish-brown, containing a brownish-green pigment that extends above the hymenium and turns red in N; paraphyses pigmented, slightly swollen, with bluish-green pigment, distinctly septate in the upper part	***M. incisa***
–	Thallus inconspicuous, absent or only evident around the apothecia; apothecial disc plane, smooth; margin relatively thick and fissured, with each cracked lobe ranging from slightly sinuate to moniliform, pruinose; epithecium brown to dark brown or reddish-brown; paraphyses apices swollen, brown	***M. crenulata***
13	Apothecial disc brown to slightly orange, sometimes appearing pale brown to dirty brown, white pruinose; epithecium gray-brown to pale brown or brown, containing granules (pol+), granules insoluble in K, but rapidly soluble in N	***M. hagenii***
–	Apothecial disc pale, or pale yellow, often appears lead-gray due to a thick pruina; epithecium yellow to yellow-orange, containing granules (pol+), granules insoluble in N and K	***M. juniperina***
14	The apothecial disc slightly to thick pruina	**15**
–	The apothecial disc lacks pruina	**22**
15	With areolate or verrucose to areolate or verrucose thallus	**16**
–	Thallus inconspicuous or only at the base of the apothecia	**18**
16	Thallus verrucose or granular; apothecial disc brown to reddish-brown, with a thick white pruina; epithecium reddish-brown, granules insoluble in K and N; paraphyses usually brown	***M. convexa***
–	Thallus rimose-areolate, yellowish brown to red brown; apothecia black, with a slight white pruina; apothecial margin concolorous with the disc; epithecium brown to blackish-brown, or olive, granules indistinct (pol ±)	**17**
17	Thallus yellowish-brown to brown; epithecium olive, green-black, green-brown, granules slowly dissolving and turning pink to red in N, insoluble in K; paraphyses apically swollen, containing green to blue pigment	***M. altunica***
–	Thallus yellow or yellowish-white. epithecium brown to blackish-brown, granules insoluble in K and N but appearing slightly reddish-purple in N and golden-yellow in K; paraphyses not swollen at the tips, not pigmented	***M. nigrodisca***
18	Saxicolous	**19**
–	Corticolous	**20**
19	Apothecial disc black, sometimes appearing blue or bluish-black due to a dense, thick white pruina; apothecial margin relatively thick, entire to slightly undulate; epithecium with granules (pol +), insoluble in N, but dissolving in K; paraphyses sometimes moniliform (beaded), usually not pigmented	***M. complanata***
–	Apothecial disc with slight or no pruina, ink-blue; apothecial margin relatively thin, entire, rough, sometimes finely denticulate or with small granules; epithecium with granules (pol +), insoluble in K and N; paraphyses thick, apices strongly swollen, sometimes subspherical (up to 3 μm), containing reddish-brown pigment	***M. mons-nivis***
20	Thallus distinctly visible	**21**
–	Thallus poorly developed, completely invisible or indistinct, or present only at the base of apothecia	**22**
21	Thallus surface whitish to grayish-white; epithecium deep orange to reddish-brown, turning slightly reddish or intensifying in color in N, granules insoluble in K and slowly soluble in N; paraphyses apices slightly swollen to capitate (up to 3 μm)	***M. zosterae* var. *palanderi***
–	Thallus surface light gray; epithecium olive, or blackish-green to deep blackish-yellow-green, partly containing green pigment; the pigmented portion turns red (dominant) or pink to orange-red in N; granules insoluble in K and N; paraphyses apices swollen, submoniliform	***M. perpruinosa***
22	Apothecial disc covered with dense, thick white pruina, appearing brown to ink-blue or gray-blue to ice-blue; epithecium granules insoluble in N, rapidly dissolving in K; paraphyses apices slightly swollen, with pale brown pigment; spores with distinct halo	***M. wetmorei***
–	Apothecial disc without pruina or with slight pruina	**23**
23	Apothecial disc brown to reddish brown; epithecium granules slowly soluble in K and insoluble in N	***M. persimilis***
–	Apothecial disc purplish-brown, purplish-red, or black; epithecium reddish or orange, turning orange-red or reddish-brown in N, granules insoluble in N but soluble in K	***M. zosterae***

## Discussion

*Myriolecis* is a taxonomically challenging group to identify, with the vast majority of its species exhibiting significant morphological variation. As for thallus morphology, the thallus is lacking or poorly visible, growing within the substratum or under apothecia, immersed, dispersed as warts or fine granulose, thin and distinct (*M. semipallida*, *M. invadens*), slightly rimose to areolate (*M. perpruinosa*), usually distinct and continuous (*M. kunlunica*), or moderately thick (*M. incisa*). Regarding the apothecial characteristics, except for *M. fugiens*, all species have numerous apothecia. Most species have thick apothecial margins with varying degrees of crenation to cracking. All species have moderately developed amphithecia with abundant, dense algae. In terms of altitude and distribution, the species are primarily distributed across an altitudinal range of 2,800–3,800 m in the southern Tianshan Mountains and 3,800–4,200 m in the Kunlun Mountains. The two species, *M. wetmorei* and *M. convexa*, are distributed in the midsection of the southern foothills of the Tianshan Mountains and the northern edge of the Tarim Basin. This region is located in the warm temperate zone. Other commonly occurring species, *M. semipallida* and *M. invadens*, are distributed at higher elevations, mainly in the southern part of the Tianshan Mountains and the Pamir, Karakoram, and Kunlun Mountains.

In addition, in the taxonomic study of *Myriolecis*, lichen secondary metabolites played a critical role. The secondary chemistry of *Myriolecis* usually shows xanthones, sometimes accompanied by minor quantities of pannarin or gyrophoric acid, but atranorin or lichen products are not detectable (Poelt et al. 1995; Fröberg 1997; Laundon 2003; Śliwa 2007). For example, *Lecanora elenkinii* Mereschk., *L. thallophila* H.Magn., and *L. utahensis* H.Magn. were first considered members of the genus *Myriolecis* (formerly the *L. dispersa* group) in previous studies; however, they produce isousnic acid and were excluded from *Myriolecis* by Śliwa (2007). Śliwa (2007) considered that *Lecanora actophila* Wedd. and *L. populicola* (DC.) Duby both contain xanthones and suggested their placement in *Myriolecis* (formerly the *L. dispersa* group). These two species are listed in *Myriolecis* in recent studies (Nimis 2016; Cannon et al. 2022). Molecular analysis of DNA sequences is also an effective tool for species identification and is very helpful in discovering new species and understanding relationships among taxa within *Myriolecis*. More recent multilocus phylogenetic studies by Zhao et al. (2016) and Kondratyuk et al. (2019) resolved members of the *Myriolecis* branch. In this study, a relatively large number of species were included in the phylogenetic analysis. Our two-locus phylogeny (ITS and mtSSU) recovered the *Myriolecis* species within a major clade, suggesting that the genus is monophyletic. However, some internal branches of this lichen genus remain poorly resolved (bootstrap support < 50%). Future studies may require more taxon sampling and molecular markers to resolve the phylogenetic relationships among the branches of this genus.

## Conclusion

The study of lichen taxonomy in extreme environments is as much a research hotspot as studies of other biological groups. Conducting lichen taxonomic research in these regions requires a strong focus on morphological and anatomical characteristics, as well as variations in secondary metabolites. The research area of this paper covers the Kunlun, Karakoram, and central Tianshan Mountains to their southernmost tip. During the collection of specimens in these areas, it was observed that the dominant saxicolous lichen taxa, such as *Aspicilia*, *Acarospora*, *Lobothallia*, Lichinaceae, and Teloschistaceae, exhibited remarkable vitality and developed unique adaptive characteristics. From this study, it can be concluded that the Kunlun Mountains harbor a rich and understudied lichen diversity. The research findings will also provide fundamental data for the lichen flora of China and subsequent studies on adaptive evolution.

## Supplementary Material

XML Treatment for Myriolecis convexa

XML Treatment for Myriolecis incisa

XML Treatment for Myriolecis kunlunica

XML Treatment for Myriolecis fugiens

XML Treatment for Myriolecis mons-nivis

XML Treatment for Myriolecis wetmorei

## Additional information

### Artificial Intelligence (AI) use

The authors accept full responsibility for the content of the manuscript, including the disclosure of any use of AI.

No AI tools were used in the preparation of this manuscript.

### Author ORCIDs

R. Mamut https://orcid.org/0000-0001-8624-9584

### Data availability

The data used in this manuscript are available in the text and figures, and the data used for phylogenetic analysis are available in NCBI GenBank.

## Supplementary materials

Supplementary material 1 The taxa and sequences used in the two-locus dataset; newly generated sequences for this study are in bold Data type xls https://binary.pensoft.net/file/1673102This dataset is made available under the Open Database License (http://opendatacommons.org/licenses/odbl/1.0/). The Open Database License (ODbL) is a license agreement intended to allow users to freely share, modify, and use this Dataset while maintaining this same freedom for others, provided that the original source and author(s) are credited. Tursunay Payzulla, Guldiyar Adil, Reyim Mamut
